# Thyroid nodulectomy: A promising approach to the management of solitary thyroid nodules

**DOI:** 10.3892/br.2024.1805

**Published:** 2024-06-17

**Authors:** Abdulwahid M. Salih, Aso S. Muhialdeen, Deari A. Ismaeil, Yadgar A. Saeed, Hardi M. Dhahir, Hiwa O. Baba, Fahmi H. Kakamad, Abdullah A. Qadir, Marwan N. Hassan, Shko H. Hassan, Berun A. Abdalla, Mohammed S. Mohammed

**Affiliations:** 1 College of Medicine, University of Sulaimani, Sulaimani, Kurdistan 46001, Iraq; 2 Scientific Affairs Department, Smart Health Tower, Sulaimani, Kurdistan 46001, Iraq; 3 Kscien Organization for Scientific Research, Sulaimani, Kurdistan 46001, Iraq; 4 Research Center, University of Halabja, Halabja, Kurdistan 46018, Iraq

**Keywords:** thyroid nodule, enucleation, total thyroidectomy, thyroid lobectomy

## Abstract

The choice between nodulectomy and lobectomy for managing thyroid nodules is a subject of debate in the field of thyroid surgery. The present study aims to share the experience of a single center in managing solitary thyroid nodules through nodulectomy from January 2023 to October 2023. The inclusion criteria encompassed symptomatic or suspicious solitary nodules and medically necessitated cases. The extracted data included patient demographics, medical history, symptoms, diagnostic details, surgery indication, procedure outcome and histopathological findings. The follow-up included clinic visits and phone calls. The mean age of the patients was 36.64±11.63 years, with 85.0% females and 15.0% males. Predominantly, patients were housewives (58.5%). Neck swelling (62.3%) was the most common presentation. Ultrasound examination revealed mixed nodules in more than half of the cases (54.7%). Right nodulectomy was performed in 26 cases (49.1%) and left nodulectomy in 23 (43.4%), and four cases (7.5%) underwent isthmusectomy. The mean operation time was 36.04±9.37 min and no drainage tube was used in any of the cases. One case (1.9%) of seroma was the only observed complication during the observational period. Nodulectomy may be a suitable choice for managing benign, large, solitary thyroid nodules, small suspicious nodules or microcarcinomas.

## Introduction

Thyroid nodule refers to a distinct abnormality within the thyroid gland that can be differentiated from the normal thyroid tissue using ultrasound (U/S) imaging. The widespread adoption of advanced imaging techniques, such as neck scans, has resulted in increased identification of thyroid nodules necessitating medical assessment. It is worth noting that as many as 60% of adults in the general population may have one or more thyroid nodules (1). The primary concern is the potential for malignancy, yet the prevalence of cancer in unselected cohorts with thyroid nodules typically ranges from 1 to 5% (2). In cases where cancer is detected, it is often characterized by being small, confined within the thyroid gland, and having a slow and non-aggressive growth pattern (3). Benign thyroid nodules necessitating intervention are infrequent. The most common types include hyperfunctioning nodules and those causing compression of vital structures such as the trachea or esophagus, in addition to eliciting general neck discomfort and cosmetic concerns, all of which may significantly impact the quality of life (4). The choice between nodulectomy and lobectomy for managing solitary thyroid nodules is a subject of debate in the field of thyroid surgery (5). The present study aims to share the experience of a single center in managing solitary thyroid nodules through nodulectomy.

## Patients and methods

### Study design

The study was structured as a single-center retrospective descriptive study of consecutive patients treated at Smart Health Tower (Sulaimani, Iraq) from January 2023 to October 2023. All patients provided informed consent for inclusion and publication of their data (medical records, images or figures) in this study. The study was ethically approved by the ethics committee of the University of Sulaimani (Sulaimani, Iraq; approval no. 82).

### Inclusion and exclusion criteria

The following inclusion criteria were applied: i) Patients who underwent nodulectomy for symptomatic or suspicious solitary thyroid nodules; and ii) patients with documented medical conditions that required nodulectomy as a treatment option. Patients were excluded if they had incomplete medical documentation, did not give their informed consent to participate, had been confirmed to have cancer, had nodules in the posterior part of the thyroid gland or the inferior pole of the thyroid lobe near the parathyroid gland and recurrent laryngeal nerve (RLN), multinodular goiter, hypothyroidism and/or positivity for anti-thyroid peroxidase (ATPO).

### Data collection

Electronic medical records were reviewed to collect data including the patient's age, gender, occupation, medical history, symptoms, clinical examination, preoperative diagnostic imaging, laboratory results, fine needle aspiration cytology (FNAC) (6), the primary indication for nodulectomy, operation details and histopathological findings.

### Preoperative preparation

Patients scheduled for nodulectomy underwent thyroid function tests [thyroid stimulating hormone (TSH), free triiodothyronine and free thyroxine] and a neck U/S performed by an experienced radiologist. Further investigations included ATPO, thyroglobulin, complete blood count and viral screening. A mobile application was developed and installed for patients to provide preoperative preparation and postoperative guidance. The decision on nodulectomy was based on a careful assessment of patients' thyroid status, risk factors, nodule characteristics, age, tolerability for the second operation, patient consent, preoperative levels of calcitonin and thyroglobulin, and the chance to preserve thyroid function and achieve complete nodule removal. Clinically, the thyroid gland swelling was graded according to the WHO classification of goiter (7). The thyroid nodules were classified on ultrasonography based on the American College of Radiology Thyroid Imaging Reporting and Data Systems (TIRADS) classification (8).

### Surgical intervention

**Figure 1:**
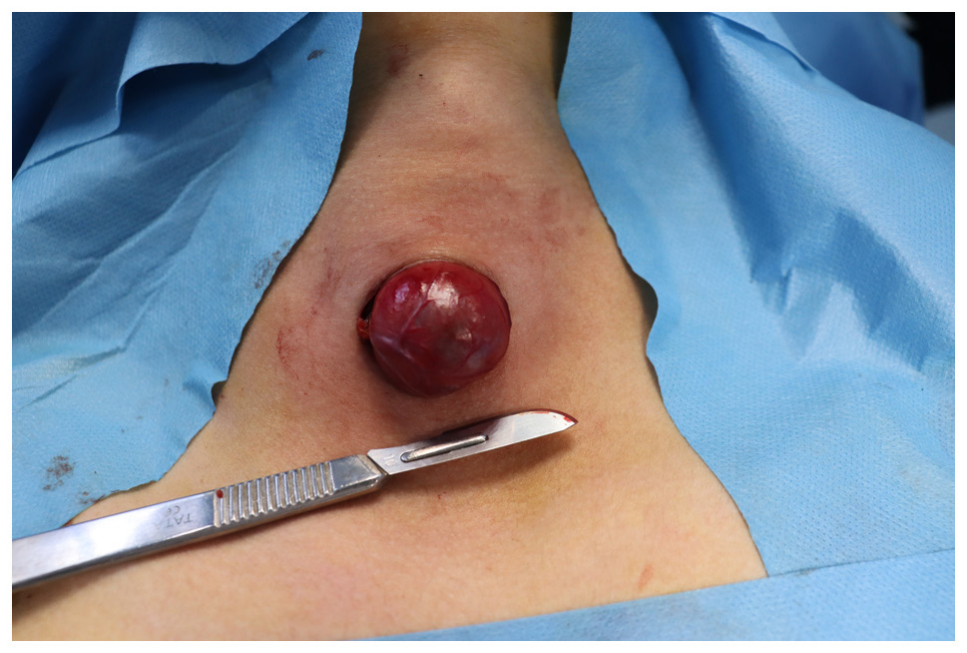
Intraoperative image shows a collar incision along a natural skin crease in the midline, which extended more to the site of the nodule.

**Figure 2:**
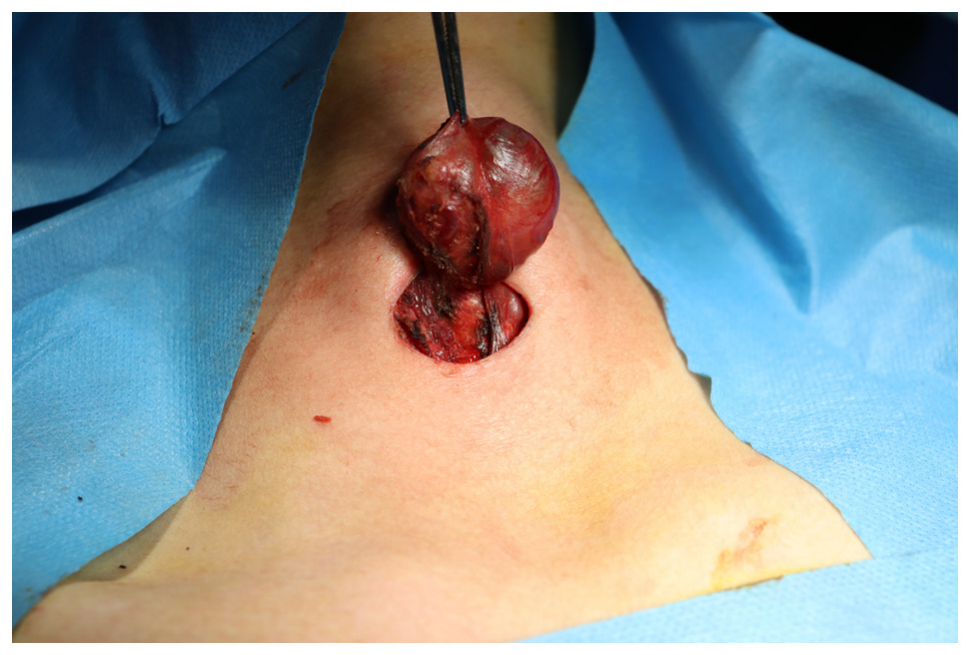
Intraoperative image shows a ligasure employed to both incise and cauterize the blood vessels and tissues while the surgeon removes the nodule.

**Figure 3:**
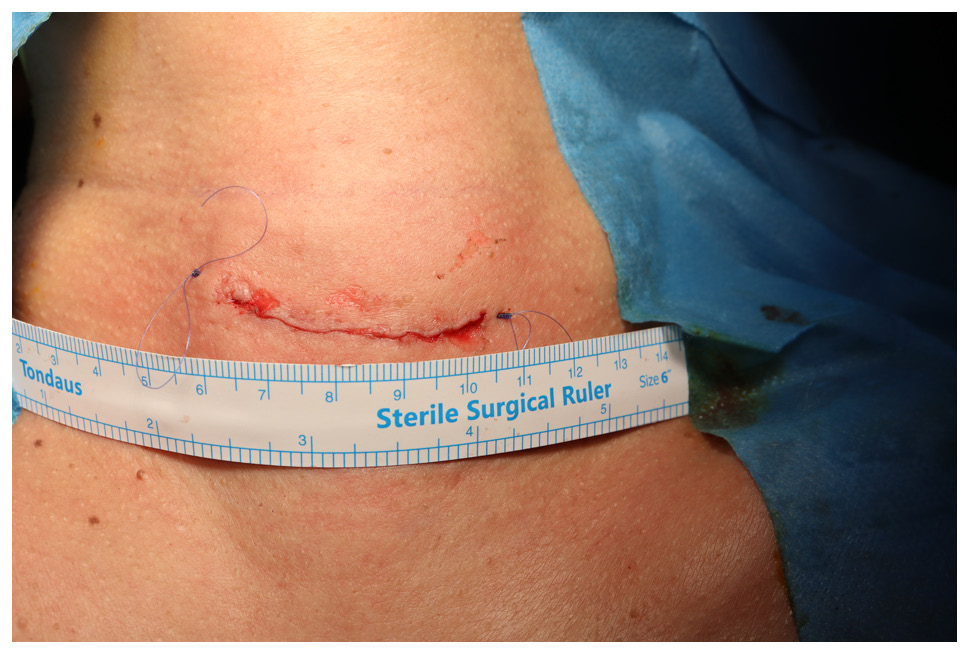
Postoperative image showing the site and length of the incision along the neck's natural crease.

Under general anesthesia, the patients were placed in a supine position with their necks extended and elevated. A collar incision was made along the natural skin crease in the midline, extending to the site of the nodule, with the contralateral extension crossing the midline (Fig. 1). The length of the incision varied from 2 to 4 cm, depending on the size of the nodule being addressed. The tissue flap was elevated on both sides below and above the specific area to expose the isthmus. The procedure aimed for the precise removal of the nodule, ensuring a 2-millimeter healthy margin of thyroid tissue covering the nodule's capsule, except for cases where the nodule was directly adherent to the thyroid capsule. Ligasure was utilized to cut and seal the blood vessels and tissues to minimize bleeding and the risk of damaging surrounding structures during the procedures (Fig. 2). Hemostasis was secured, with no drain left, followed by the closure of the surgical site in multiple layers (Fig. 3). No antibiotics were administered to patients either during or after the surgery.

### Histopathological procedure

The tissue specimen was initially placed into a tissue cassette. Subsequently, the cassettes underwent processing utilizing the DiaPath Donatello automated processor, following a standardized 11-h protocol involving immersion in alcohol, xylene and paraffin. After embedding in paraffin and trimming, the resultant blocks were sectioned (thickness, 4-6 µm) onto standard glass slides. These slides were then incubated overnight at 60˚C and subsequently stained using the DiaPath Giotto automated stainer (DiaPath), employing a 1% solution for 10 min for hematoxylin and eosin staining with Gill's II hematoxylin, according to the manufacturer's intructions. The slides were then dried and coverslips were applied. The examination was conducted using a light microscope (Leica Microsystems GmbH).

### Follow-up

After the operation, patients had a follow-up appointment one week later, followed by ongoing phone calls every week for up to one month. Patients who developed surgical site infections or seromas were scheduled for clinic visits.

### Statistical analysis

The data were extracted into an Excel sheet (2019; Microsoft Corp.). Descriptive data analysis was performed using the Statistical Package for the Social Sciences version 25 (IBM Corp.). The results were presented as means, standard deviations, frequencies and percentages.

## Results

### Demographic and clinical characteristics

The study included a total of 53 patients. The mean age was 36.64±11.63 years, with a range of 18-55 years. In total, 85.0% of the patients were female and 15.0% were male. The majority of the patients were housewives (58.5%). In terms of smoking status, 1.9% were active smokers. The most common clinical presentation was neck swelling (62.3%). The clinical examination revealed grade 2 swelling in 41.5% of cases and an equal percentage demonstrated a firm-to-hard consistency of the thyroid (Table I).

### Diagnostic findings

The mean TSH level was 1.5±1.29 mIU/l. The U/S findings showed that more than half of the cases (54.7%) had a mixed texture of the nodule. Regarding the TIRAD classification, 71.7% were TIRAD grade (TR)3, 13.2% were TR4 and 11.3% were TR2. The affected lobe was the right one in 49.1% of cases and the left in 43.4%. The most common FNAC finding among tumors (among those 42 cases had FNAC examination) was Bethesda II in 52.8% of the cases and the least common was Bethesda VI (5.6%). Thyroid function tests were within normal reference ranges in most of the cases (83.0%). The main indication for intervention was a large nodule (69.9%) (Table II).

### Operation

A total of twenty-six cases (49.1%) underwent right nodulectomy, 23 cases (43.4%) underwent left nodulectomy and four cases (7.5%) underwent isthmusectomy. The mean duration of the operations was 36.04±9.37 min. No drainage tube was used in any of the cases. The incision was made on the neck crease in 58.5% of the cases. The mean incision length was 4.0±0.83 cm (Table III). In the majority of cases (91.3%), the nodules were situated at the junction of the isthmus and the lobe (data not shown). Consequently, during the procedures, a portion of the isthmus was excised. There was no postoperative complication, except for one case of seroma (1.9%) (Table III).

### Histopathological findings

Histopathological examination revealed a hyperplastic thyroid nodule with no malignancy in 52.8% of cases, a follicular adenoma in 18.9%, an adenomatoid nodule with oncocytic cell changes in 9.4%, a minimally invasive carcinoma in 13.2% (papillary thyroid microcarcinoma 7.5% and follicular thyroid carcinoma with capsular invasion 5.7%) and a simple colloid cyst in 1.9%. Patients with minimally invasive follicular thyroid carcinoma (5.7%) and papillary thyroid microcarcinoma (7.5%) consequently underwent total thyroidectomy.

## Discussion

Thyroid nodules exhibiting characteristics such as hardness, fixation or rapid growth necessitate immediate assessment. Studies have established that the specific location of the nodule within the thyroid gland is an independent factor contributing to the risk of malignancy. Nodules originating in the isthmus region have the highest likelihood of malignancy, while those situated in the lower portion of the thyroid lobes have the lowest risk when compared to those in the middle or upper regions (9,10). In the current study, upon examination, it was found that tumors in 35.9% of cases exhibited a firm consistency, while 22.6% were hard and 41.5% were in between. Tumors in only 7.5% of the cases involved the isthmus.

The primary method used for thyroid imaging is U/S. To assess thyroid nodules and preliminarily estimate their potential for malignancy, the radiologist should consider several factors, including hypoechogenicity, infiltrative, lobulated or irregular margins, the presence of microcalcifications and a shape that is taller than it is wide. Alongside evaluating the nodule, U/S examination should comprehensively examine all cervical lymph node areas and any suspicious lymph nodes should be documented (11). The ability to accurately distinguish malignant lesions from benign ones varies among these features, and none of them have consistently provided reliable differentiation between the two (12). In the present study, U/S revealed a solid texture in 39.6% of the cases, with 71.7% categorized as TR3.

In general, FNAC represents the subsequent phase in the evaluation of a thyroid nodule. This procedure should be selectively performed for nodules deemed highly suspicious based on both U/S and clinical assessments. The outcomes of this cytological analysis have a significant role in refining subsequent treatment strategies (13). Nevertheless, various potential diagnostic challenges exist that may result in erroneous outcomes, such as false positives, false negatives, indeterminate findings or non-diagnostic results (14). It is noteworthy that a substantial portion, up to 42%, of cases may fall into the indeterminate category, encompassing both follicular neoplasms and those with suspicious malignancy features (15). The predominant FNAC results among the patients of the present study were Bethesda II in 52.8% of cases and Bethesda IV in 15.1%.

Surgical intervention is a viable option for hyperfunctioning nodules and nodules causing compression, although there are various minimally invasive alternatives. These encompass U/S-guided ablation techniques such as percutaneous ethanol injection, the application of thermal energy in the form of laser, radiofrequency, high-intensity focused US or microwave energy. Radiofrequency and laser ablations have demonstrated notable efficacy in reducing nodule volumes (16). When surgical intervention is deemed necessary, the determination of the appropriate extent of resection is contingent upon several factors. These factors encompass the presence of symptoms, the existence of nodules on the contralateral side, the thyroid's functional status, concurrent medical conditions, familial medical history, surgical risks and the patient's preferences (4). Surgical intervention was warranted in the present cases primarily due to the presence of large nodules in 69.9% of cases, toxic nodules in 15.0% and suspicion of follicular neoplasms in 7.5%.

When malignancy is suspected, the least extensive surgical procedure typically considered is lobectomy along with isthmusectomy. In rare instances, isthmusectomy alone could also be considered (4). Certain studies suggest the use of minimally invasive US-guided ablation methods as an alternative to surgery for managing small nodules that raise suspicion (17). Advancements in surgical technology, including thermal sealing, have led to substantial modifications in surgical procedures. These advancements enable the safe removal of nodules along with an appropriate margin of healthy tissue for diagnostic purposes in a bloodless manner, without the requirement for extensive manipulation and ligation of major blood vessels (5). Recent research has revealed that the size of the thyroid remnant volume can impact the occurrence of postoperative hypothyroidism (18). In their study involving 186 patients who underwent unilateral lobectomy, De Carlucci *et al* (18) and others discovered that the occurrence of hypothyroidism was 6.3 times higher when the US-measured volume of the thyroid remnant was 4 ml or less (19).

It has been shown that the absence of hypoechogenic attenuation, irregular margins, or microcalcifications as identified through U/S holds a notably high negative predictive value for excluding malignancy in Bethesda Ⅲ nodules. Consequently, in such cases, nodulectomy emerges as an appealing option (20). Findings from a particular study have shown that when undertaken for the specified indications, minimally invasive thyroid nodulectomy results in a notable reduction in the post-operative risk of hypothyroidism in comparison to both formal open lobectomy and minimally invasive lobectomy (5). In the current study, there was no reported case of hypothyroidism following the surgery and there was only one case of seroma as a complication of the procedure. Seroma formation is frequently associated with procedures such as mastectomy, laparoscopic inguinal hernia repair, axillary lymphadenectomy and abdominoplasty. However, it is less commonly reported following thyroid surgery, with an incidence ranging from 1.3 to 7%. In the majority of cases, seromas resolve spontaneously and do not require intervention (21). Histopathological examination revealed three cases of minimally invasive follicular thyroid carcinoma and four cases of minimally invasive papillary thyroid microcarcinoma. All of these cases underwent total thyroidectomy. Due to limited data on nodulectomy being available, it was not possible to further compare nodulectomy with lobectomy and other procedures.

Different surgical techniques have different purposes and are appropriate in certain situations; understanding these distinctions is critical for both patients and healthcare practitioners. Nodulectomy may reduce the probability of postoperative hypothyroidism by maintaining a significant portion of the thyroid gland (5). This procedure may typically demand less time in comparison to total thyroidectomy or lobectomy. It may carry a low likelihood of complications, including RLN injury and parathyroid gland damage, when compared with more extensive surgical interventions. The present study has several limitations. These include the small sample size, short follow-up duration and reliance on data from a single center, which limit the generalizability of the results. Future studies with a sufficient sample size and robust study design are required to better evaluate this procedure and compare it with other available management techniques.

In conclusion, nodulectomy may be a suitable choice for managing benign large nodules, small suspicious nodules or microcarcinomas, and for situations where preserving thyroid function is paramount. However, the definitive outcome of the procedure requires much more intensive investigation. It needs to be compared in clinical trials with other management techniques such as lobectomy, isthmusectomy or U/S-guided ablation methods.

## Figures and Tables

**Table I:** Demographic data and clinical characteristics of patients (n=53).

Item	Value
Age, years	36.64±11.63
Sex	
Female	45 (85.0)
Male	8 (15.0)
Occupation	
Housewife	31 (58.5)
Worker	2 (3.8)
Student	6 (11.3)
Teacher	5 (9.4)
Other	9 (17.0)
Marital status	
Single	11 (20.8)
Married	42 (79.2)
Past medical history	
Thyroid disease	1 (1.9)
Hypertension	1 (1.9)
Negative	51 (96.2)
Past surgical history	
Breast operation	1 (1.9)
Negative	53 (98.1)
Family history	
Cancer of the liver, stomach	1 (1.9)
Negative	53 (98.1)
Smoking	
Active	1 (1.9)
Passive	6 (11.3)
Negative	46 (86.8)
Presentation	
Neck pain	1 (1.9)
Neck swelling	33 (62.3)
Weakness	3 (5.6)
Thyroid checkup	7 (13.2)
Thyroid problem	9 (17.0)
Grade of swelling	
G0	10 (18.9)
G1	15 (28.3)
G1-2	6 (11.3)
G2	22 (41.5)
Thyroid consistency upon examination	
Firm	19 (35.9)
Firm-hard	22 (41.5)
Hard	12 (22.6)

Values are expressed as the mean ± standard deviation or n (%).

**Table II:** Diagnostic findings and indications of surgery.

Item	Value
TSH, µIU/ml	1.5±1.29
Ultrasound findings (nodule texture)	
Cystic	1 (1.9)
Homogenous	1 (1.9)
Mixed	29 (54.7)
Solid	21 (39.6)
Not mentioned	1 (1.9)
TIRAD classification	
TR2	6 (11.3)
TR3	38 (71.7)
TR4	7 (13.2)
N/A	2 (3.8)
Involved lobe	
Left	23 (43.4)
Right	26 (49.1)
Isthmus	4 (7.5)
Calcification	
Yes	8 (15.0)
No	45 (85.0)
Retrosternal extension	
Yes	1 (1.9)
No	53 (98.1)
Fine needle aspiration result	
Bethesda I	2 (3.8)
Bethesda II	28 (52.8)
Bethesda IV	8 (15.1)
Bethesda V	1 (1.9)
Bethesda VI	3 (5.6)
N/A	11 (20.8)
Thyroid state	
Euthyroid	44 (83.0)
Hyperthyroid	9 (17.0)
Indication for surgery	
Large nodule	37 (69.9)
Toxic nodule	8 (15.0)
Radiologically suspicious nodule	2 (3.8)
Follicular neoplasm	4 (7.5)
PTC	2 (3.8)

Values are expressed as the mean ± standard deviation or n (%). TSH, thyroid stimulating hormone; TIRAD, thyroid imaging reporting and data system; PTC, papillary thyroid carcinoma; N/A, not available.

**Table III:** Details of the operations and histopathological findings.

Variable	Value
Treatment	
Left nodulectomy	23 (43.4)
Right nodulectomy	26 (49.1)
Isthmusectomy	4 (7.5)
Duration, min	36.04±9.37
Drain	
Yes	0 (0)
No	53(100)
Incision on the crease	
Yes	31 (58.5)
No	22 (41.5)
Incision length, cm	4.0±0.83
Complications	
Hypothyroidism	0 (0)
Infection	0 (0)
Seroma	1 (1.9)
Hematoma	0 (0)
RLN injury	0 (0)
Histopathological findings	
PTMC	4 (7.5)
Minimally invasive FTC with capsular invasion	3 (5.7)
Hyperplastic thyroid nodule with no malignancy	28 (52.8)
Follicular adenoma	10 (18.9)
Adenomatoid nodule with oncocytic cell changes with no malignancy	5 (9.4)
N/A	2 (3.8)
Simple colloid cyst with benign thyroid tissue with no malignancy	1 (1.9)

Values are expressed as the mean ± standard deviation or n (%). RLN, recurrent laryngeal nerve; PTMC, papillary thyroid microcarcinoma; FTC, follicular thyroid carcinoma; N/A, information not available.

## Data Availability

The data generated in the present study are included in the figures and/or tables of this article.
